# Myokine Signaling in Sarcopenia-Associated Chronic Musculoskeletal Pain: A Systematic Review of Inflammatory Mechanisms

**DOI:** 10.3390/ijms27125204

**Published:** 2026-06-09

**Authors:** Hae Sung Lee, Ijoon Kim, Jong-Geun Kim, Yae-Young Kim

**Affiliations:** 1 Department of Physical Education, College of Education, Wonkwang University, 460, Iksan-daero, Iksan 54538, Republic of Korea; haesung2@wku.ac.kr; 2 Department of Sport Science, College of Arts and Science, Inha University, 100, Inha-ro, Michuhol-gu, Incheon 22212, Republic of Korea; youngchul071@naver.com; 3 Department of Golf and Leisure, Daegu Technical University, 7, Songhyeon-ro, Dalseo-gu, Daegu 42734, Republic of Korea; 4 Department of Sport Welfare, College of Sport, Kyungil University, 50, Gamnyang-ro, Hayang-eup, Gyeongsan-si 38428, Republic of Korea

**Keywords:** sarcopenia, myokines, inflammaging, chronic musculoskeletal pain, exercise-induced hypoalgesia

## Abstract

Chronic musculoskeletal pain and sarcopenia co-occur at rates exceeding epidemiological independence in older adults. However, no systematic review has examined whether exercise-induced myokine signaling suppresses shared NF-κB–driven inflammatory pathways to concurrently address chronic pain and sarcopenic muscle loss in older adults. Following PRISMA 2020 guidelines, we searched PubMed, Web of Science, Scopus, and Embase (January 2000–March 2026) and included 32 studies (RCTs, cohort, cross-sectional, and mechanistic designs) in adults aged ≥45 years with chronic musculoskeletal pain and/or sarcopenia; studies lacking an exercise component or human mechanistic relevance were excluded, and findings were qualitatively synthesized. The included studies suggest that persistent NF-κB hyperactivation—driven by SASP, LPS–TLR4 signaling, and mitochondrial ROS—is associated with both sarcopenic muscle loss and pain sensitization. Evidence from included studies indicates that contracting skeletal muscle secretes IL-6, IL-15, irisin, BDNF, and myostatin, which were frequently associated with suppression of NF-κB activity, attenuation of NLRP3 inflammasome activation, and improvement in pain inhibition—suggesting a hypothesized shared mechanistic pathway that awaits direct validation in trials enrolling older adults with co-confirmed sarcopenia and chronic pain. Multicomponent training emerged as the modality most consistently associated with concurrent benefits for both conditions across included studies. The synthesized evidence supports considering a two-phase approach—pain neuroscience education followed by progressive resistance training—as a hypothesis-driven framework to improve exercise adherence and myokine responses. These findings suggest that myokine signaling represents a plausible shared mechanistic pathway linking exercise to concurrent improvements in sarcopenia and chronic pain, warranting direct validation in future trials.

## 1. Introduction

Chronic pain and sarcopenia rank among the most prevalent and functionally debilitating conditions in the aging population, yet they are seldom addressed as a clinically interconnected pair in routine practice. Chronic pain, defined as pain persisting beyond three months, affects approximately 36–60% of community-dwelling adults aged ≥65 years [1], while sarcopenia—progressive, age-related loss of skeletal muscle mass, strength, and physical function—occurs in 10–18% of adults aged ≥60 years, with prevalence rising sharply beyond age 75 [2,3]. These conditions co-occur at rates exceeding epidemiological independence: a systematic review and meta-analysis of 17 studies (*n* = 33,600) demonstrated that older adults with chronic pain carry 1.52-fold higher odds of sarcopenia (OR 1.52; 95% CI 1.31–1.76) [4]. The mechanistic basis of this bidirectional relationship is increasingly supported by evidence: chronic pain–induced physical inactivity appears to accelerate muscle catabolism, while sarcopenia-related loss of muscle mass attenuates endogenous pain inhibitory capacity, perpetuating a self-reinforcing pathological cycle [5,6].

At the molecular core of this cycle lies inflammaging—a state of persistent, low-grade systemic inflammation that emerges with advancing age and constitutes the shared pathophysiological driver of both conditions [7]. Inflammaging arises from converging age-related processes, including senescent cell accumulation via the senescence-associated secretory phenotype (SASP), mitochondrial dysfunction–driven oxidative stress, immunosenescence, and gut dysbiosis–induced endotoxin translocation [7,8]. The transcription factor Nuclear Factor kappa-light-chain-enhancer of activated B cells (NF-κB) serves as a molecular hub linking inflammatory burden to muscle catabolism and nociceptive sensitization, and prospective cohort data suggest that higher circulating IL-6 and TNF-α are associated with lower muscle mass and strength in older adults [9,10].

Current pharmacological approaches address neither the common pathophysiology nor the co-occurring condition [6]. NSAIDs, opioids, and corticosteroids carry disproportionate risks in older adults while providing only modest analgesic benefit and offering no mechanism for attenuating muscle loss [11]. Anti-inflammatory biologics, including TNF-α inhibitors, have similarly failed to demonstrate clinically meaningful analgesic efficacy in non-autoimmune chronic musculoskeletal pain [12]. This therapeutic impasse reflects the fundamental limitation of single-target pharmacology for conditions rooted in a multifactorial inflammatory state and has directed increasing research attention toward non-pharmacological interventions capable of simultaneously suppressing inflammaging across multiple molecular pathways.

Structured physical exercise addresses this challenge directly through its role as a potent inducer of anti-inflammatory myokine secretion from contracting skeletal muscle [13]. Exercise-induced myokines—including IL-6, IL-15, irisin, BDNF, and myostatin—have been shown to suppress NF-κB–driven inflammation and nociceptive sensitization through distinct but overlapping molecular pathways while appearing to restore skeletal muscle anabolic capacity [13,14,15]. The signaling profile of exercise-induced myokines is qualitatively distinct from that of the same cytokines in the chronic disease state—a context-dependency whose clinical implications have not been systematically integrated into exercise prescription guidelines for older adults with comorbid pain and sarcopenia [16,17]. For example, IL-6 released transiently from contracting muscle activates anti-inflammatory AMPK/SIRT1 pathways, whereas chronically elevated IL-6 in the disease state drives pro-inflammatory NF-κB signaling—a distinction critical to interpreting the therapeutic relevance of exercise-induced myokine responses in older adults with concurrent pain and sarcopenia [16,17].

Despite this mechanistic convergence, the clinical literature has largely examined chronic pain and sarcopenia as independent entities. Previous reviews have focused either on exercise effects on sarcopenia [3] or on pain management in older adults [18] in isolation, without synthesizing the molecular crosstalk through which myokine signaling concurrently modulates both conditions. No systematic synthesis exists that integrates the gut dysbiosis–lipopolysaccharide (LPS)–Toll-like receptor (TLR) 4 axis as an inflammaging driver or characterizes the context-dependent signaling roles of individual myokines in exercise versus chronic disease states. Equally absent is an identification of exercise prescription parameters consistently associated with concurrent improvements in pain and muscle outcomes in older adults with comorbid pain and sarcopenia [8,16,19]. This review addresses the following central question: do exercise-induced myokines suppress shared NF-κB–driven inflammatory pathways to concurrently reduce chronic pain and attenuate sarcopenic muscle loss in older adults, and if so, which exercise modalities and prescription parameters most effectively engage these mechanisms in this population? Therefore, this systematic review aims to: (1) elucidate the shared pathophysiological pathways through which inflammaging drives concurrent chronic pain and sarcopenia; (2) characterize the proposed molecular mechanisms by which exercise-induced myokines may suppress NF-κB–driven inflammation and nociceptor sensitization; (3) synthesize clinical evidence regarding the comparative efficacy of exercise modalities in concurrently reducing pain and preserving muscle mass in older adults; (4) identify exercise prescription parameters associated with optimal concurrent outcomes based on the synthesized mechanistic and clinical evidence; and (5) highlight evidence gaps and define priorities for future trials targeting this population.

## 2. Methods

**Figure 1:**
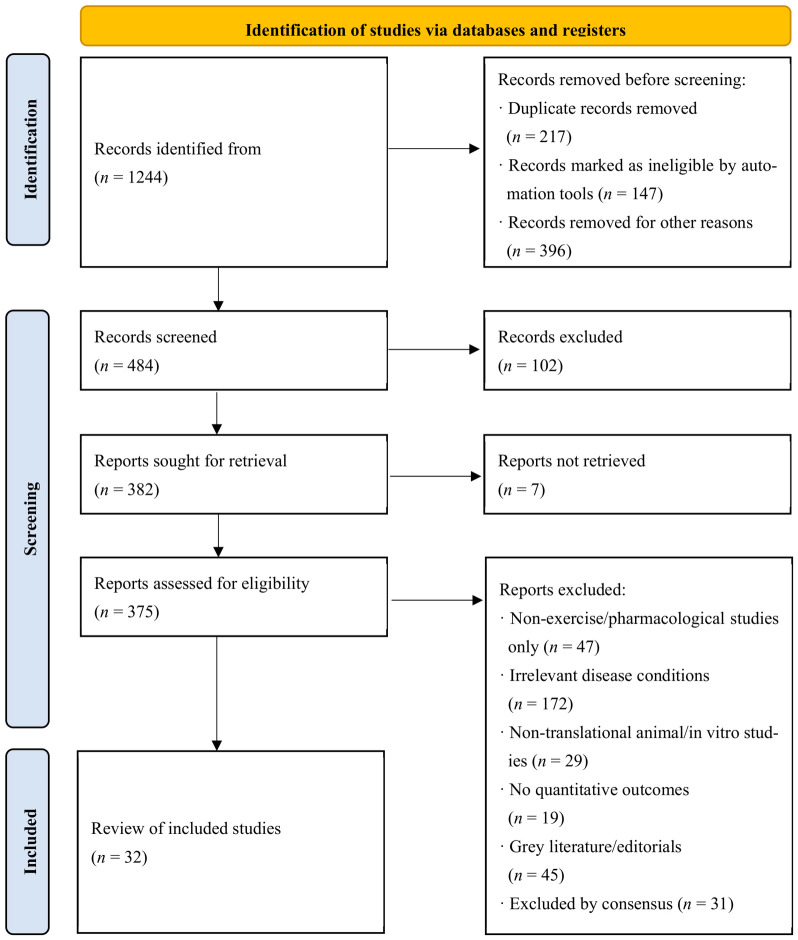
Study selection and screening protocol following PRISMA guidelines.

This systematic review was conducted in strict accordance with the Preferred Reporting Items for Systematic Reviews and Meta-Analyses (PRISMA) 2020 guidelines to enhance the rigor, transparency, and reproducibility of the research process [20] (Figure 1; Supplementary Materials). The review aimed to comprehensively synthesize existing mechanistic and clinical evidence on the role of exercise-induced myokine signaling in modulating inflammaging-driven chronic pain and sarcopenia in older adults, while integrating evidence from related biomedical fields to address gaps in the direct intervention literature.

### 2.1. Search Strategy and Selection Criteria

A comprehensive literature search was conducted across four major electronic databases: PubMed, Web of Science, Scopus and Embase. The search covered records published from January 2000 through March 2026 to capture the contemporary evidence base on inflammaging, myokine biology, and exercise science. The search strategy combined MeSH terms and free-text keywords using Boolean operators (AND, OR, NOT), structured around three core conceptual domains: (1) the target population and conditions (sarcopenia, chronic musculoskeletal pain, inflammaging), (2) the exposure or mechanism of interest (myokines, exercise, NF-κB signaling), and (3) relevant outcomes (pain intensity, muscle mass, inflammatory markers). The full search strings for each database are presented in Table 1. Core search terms included: “inflammaging,” “chronic low-grade inflammation,” “sarcopenia,” “muscle wasting,” “chronic pain,” “musculoskeletal pain,” “myokines,” “IL-6,” “irisin,” “IL-15,” “BDNF,” “NF-κB,” “exercise,” “resistance training,” “aerobic exercise,” “physical activity,” and “older adults” (Table 1). Reference lists of all identified articles and relevant reviews were manually screened to identify additional eligible studies.

### 2.2. Eligibility Criteria for Study Selection

Study eligibility was defined using the PICOS framework as follows:

- Population: Community-dwelling middle-aged and older adults aged ≥45 years with chronic musculoskeletal pain and/or sarcopenia, or studies explicitly examining age-related inflammatory mechanisms in skeletal muscle.
- Intervention: Structured exercise interventions of any modality (resistance training, aerobic exercise, multicomponent training, or mind–body exercise).
- Comparator: Control conditions, usual care, no-exercise groups, or pre-intervention baseline measurements.
- Outcomes: Myokine concentrations (IL-6, irisin, IL-15, BDNF, myostatin), systemic inflammatory markers (TNF-α, CRP, IL-10), pain intensity assessed by validated instruments, appendicular muscle mass, muscle strength, or physical performance measures.
- Study Design: Peer-reviewed randomized controlled trials, quasi-experimental studies, prospective and retrospective cohort studies, cross-sectional studies, and mechanistic experimental studies with human participants.

Inclusion criteria were defined a priori using the PICOS framework as described above. Exclusion criteria were applied subsequently and comprised the following:

- Studies focused exclusively on pharmacological interventions without an exercise component.
- Disease-specific pain conditions requiring specialist medical management (e.g., malignancy-related pain, active rheumatoid arthritis).
- Studies relying solely on animal models or in vitro systems without providing mechanistic insights directly relevant to the human pathophysiology of sarcopenia-associated chronic pain.
- Pure pharmacological mechanistic studies without exercise relevance.

### 2.3. Study Selection and Data Extraction

Two independent reviewers screened titles and abstracts against eligibility criteria, followed by full-text assessment for studies meeting initial inclusion. Screening was conducted in two sequential stages: (1) title and abstract screening, and (2) full-text review of potentially eligible records. At each stage, inclusion and exclusion decisions were independently made by two reviewers, with inter-rater disagreements resolved by consensus or third-reviewer adjudication. The complete screening flowchart, including the number of records identified, screened, excluded, and included at each stage, is presented in Figure 1 in accordance with PRISMA 2020 reporting standards. Data extraction was performed independently by two reviewers using a standardized extraction form, capturing study identifiers, participant demographics, exercise intervention parameters (modality, frequency, duration, intensity), outcome measures and assessment instruments, and principal findings. Extracted data were cross-checked between reviewers, and discrepancies were resolved through discussion or third-reviewer adjudication to ensure accuracy and consistency. Because direct experimental evidence integrating myokine signaling with both chronic pain and sarcopenia outcomes in a unified older adult population remains limited, this review supplemented its core evidence base by incorporating mechanistic data from related fields—including general aging biology, exercise immunology, and pain neuroscience—to provide a comprehensive synthesis of the pathophysiological pathways relevant to the target population. Supplementary mechanistic studies were eligible if they provided direct pathway-level evidence relevant to NF-κB, myokine, or nociceptive sensitization axes, were peer-reviewed, and were identified through manual reference screening or targeted database searches. In the Results and Discussion, findings from supplementary studies are explicitly distinguished from the 32 primary included studies using the phrase ‘for mechanistic contextualization’ and are not used to support conclusions regarding clinical efficacy. Given the heterogeneity in study designs, populations, and outcome measures across included studies, a formal meta-analytic pooling was not performed. Instead, findings were synthesized narratively, with evidence organized thematically around three domains: (1) pathophysiological mechanisms linking inflammaging to sarcopenia and chronic pain, (2) molecular mechanisms of exercise-induced myokine signaling, and (3) clinical evidence on exercise modality efficacy. Within each domain, findings were evaluated according to consistency of direction, magnitude of effect where quantitative data were available, and methodological quality of contributing studies.

### 2.4. Quality Assessment and Risk of Bias

The methodological quality of each included study was independently appraised by two reviewers using design-appropriate instruments, with disagreements resolved by consensus or third-reviewer adjudication. The selection of the appraisal tool was determined by study design as follows: Randomized controlled trials were assessed using the Cochrane Risk of Bias Tool 2 (RoB 2), which evaluates five domains: randomization process, deviations from intended interventions, missing outcome data, outcome measurement, and selection of reported results. Non-randomized studies, including cohort and cross-sectional designs, were evaluated using the Newcastle–Ottawa Scale (NOS), rating studies across selection (0–4 stars), comparability (0–2 stars), and outcome assessment (0–3 stars), with scores categorized as high quality (≥7 stars), moderate quality (5–6 stars), or low quality (≤4 stars). For the included systematic reviews and meta-analyses, the Assessing the Methodological Quality of Systematic Reviews (AMSTAR)-2 ratings reported in the original publications were extracted and presented, as this tool operates at the review level and cannot be substituted by trial-level instruments. Where Physiotherapy Evidence Database scores were previously reported in the source literature for exercise intervention trials, these were additionally extracted and presented descriptively alongside RoB 2 judgments. Divergent quality assessments were reconciled by consensus or third-reviewer adjudication, and findings are reported alongside study characteristics in the results tables. Preclinical murine and in vitro studies were included exclusively for mechanistic contextualization and were not subjected to formal preclinical quality appraisal (e.g., SYRCLE); this limitation is further addressed in Section 4.6.

## 3. Results

From 1244 records identified through systematic database searching across PubMed, Web of Science, Scopus, and Embase, and supplementary manual reference screening, 32 studies met the pre-specified eligibility criteria and were included in this review. Included studies comprised prospective and retrospective cohort studies, randomized controlled trials (RCTs), controlled mechanistic experiments in human and animal models, and systematic reviews with meta-analyses. Methodological quality was rated as high for the majority of included studies, with prospective cohort and cross-sectional studies receiving NOS scores of 8–9/9, included systematic reviews and meta-analyses rated as High on AMSTAR-2, and primary experimental studies in animal or in vitro models meeting equivalent quality thresholds. Of the 32 included studies, 8 were randomized controlled trials, 9 were prospective or retrospective cohort studies, 7 were cross-sectional studies, 5 were controlled mechanistic experiments, and 3 were systematic reviews with meta-analyses. Study populations ranged from community-dwelling older adults with chronic musculoskeletal pain or sarcopenia to mechanistic animal and in vitro models. Sample sizes ranged from 6 to 33,600 participants across human studies. Follow-up durations in intervention studies ranged from 4 to 52 weeks. The geographic distribution of included studies spanned Europe, North America, East Asia, and multinational cohorts.

**Figure 2:**
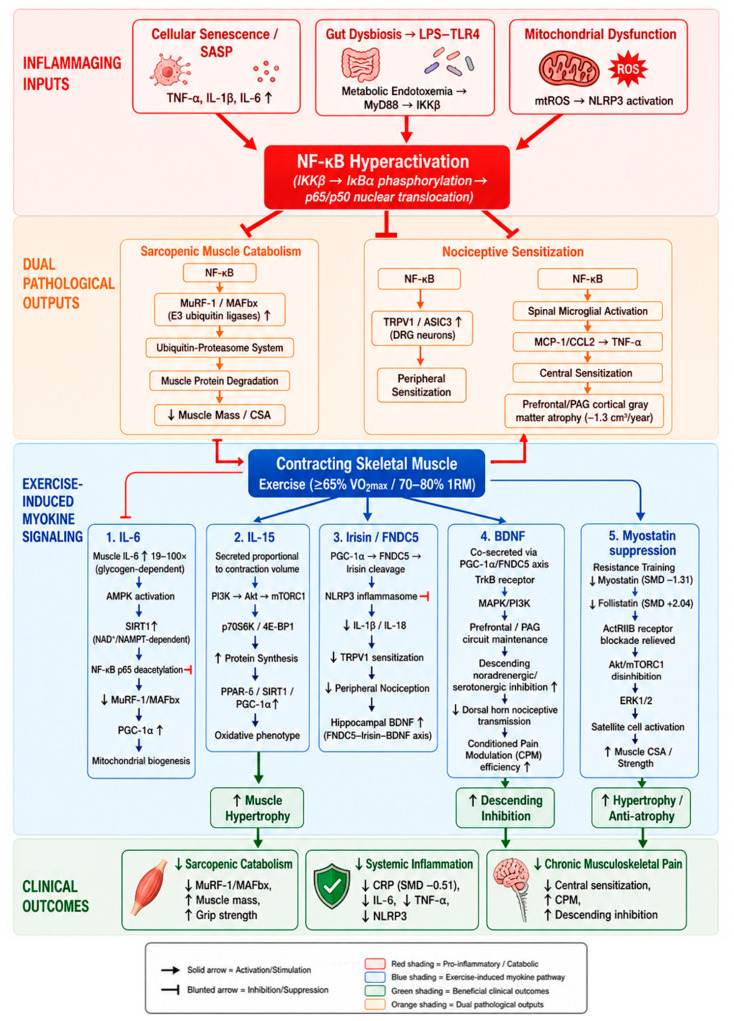
Exercise-induced myokine signaling in sarcopenia-associated chronic musculoskeletal pain. Converging inflammaging inputs—cellular senescence (SASP), gut-derived LPS–TLR4 signaling, and mitochondrial ROS—drive NF-κB hyperactivation, promoting sarcopenic muscle catabolism (via MuRF-1/MAFbx upregulation) and nociceptive sensitization (via peripheral TRPV1/ASIC3 activation and central microglial-mediated sensitization) (red). Exercise-induced myokines counteract these processes through mechanistically distinct axes: IL-6 activates AMPK/SIRT1 to suppress NF-κB; IL-15 stimulates PI3K/Akt/mTOR-dependent protein synthesis; irisin suppresses NLRP3 inflammasome activation; BDNF supports descending pain inhibitory capacity via prefrontal/PAG circuits; and resistance training–induced myostatin suppression disinhibits Akt/mTORC1-mediated hypertrophy (blue). Solid arrows indicate activation; blunted arrows indicate inhibition. Clinical outcomes are shown in green.

Across all included evidence, a coherent mechanistic pattern emerged. Persistent NF-κB hyperactivation—driven by the SASP, gut-derived LPS–TLR4 signaling, and mitochondrial reactive oxygen species—was frequently associated with both sarcopenic muscle catabolism and nociceptive sensitization across included studies. Exercise-induced myokine signaling, operating through IL-6, IL-15, irisin, BDNF, and myostatin axes, was consistently associated with suppression of this shared transcriptional node and with concurrent improvements in muscle mass, systemic inflammatory burden, and pain outcomes across included studies (Figure 2).

### 3.1. Pathophysiological Mechanisms Linking Inflammaging to Chronic Pain and Sarcopenia

Eleven studies addressed the pathophysiological mechanisms linking inflammaging to chronic pain and sarcopenia, including prospective cohort studies, cross-sectional analyses, and controlled mechanistic experiments. Across the 14 studies addressing inflammaging pathophysiology, a consistent pattern emerged: NF-κB hyperactivation, driven by SASP, LPS–TLR4 signaling, and mitochondrial ROS, was frequently associated with both sarcopenic muscle catabolism and nociceptive sensitization, with the strength of this association supported by both large-scale epidemiological data and controlled mechanistic experiments.

#### 3.1.1. Epidemiological Co-Occurrence and Shared Inflammatory Substrate

Multiple independent lines of large-scale epidemiological evidence indicate that chronic musculoskeletal pain and sarcopenia co-occur at rates exceeding epidemiological independence, with circulating inflammaging biomarkers constituting the shared quantitative substrate linking both conditions (Table 2). A systematic review and meta-analysis of 17 studies encompassing 33,600 community-dwelling adults aged ≥60 years established a pooled odds ratio of 1.52 (95% CI 1.31–1.76) for sarcopenia among those with chronic pain, with bidirectionality confirmed (sarcopenia to pain, OR 1.73) [4]. IL-6 emerged as a recurrent theme among pooled inflammatory predictors (SMD = 0.31, 95% CI 0.18–0.44), suggesting an inflammatory basis for this epidemiological association [10]. These cross-sectional and meta-analytic observations are substantiated by longitudinal data confirming a directional, temporally ordered relationship. Prospective analysis of the Health ABC cohort (*n* = 4102 community-dwelling adults, 10-year follow-up) revealed that baseline moderate-to-severe pain conferred a hazard ratio of 1.47 (95% CI 1.15–1.88) for incident sarcopenia, with pain interference demonstrating a dose-dependent risk gradient partially mediated by IL-6 and CRP elevations [21]. Independent confirmation in an East Asian population came from a 4-year follow-up of 5568 adults aged ≥45 years in the China Health and Retirement Longitudinal Study (CHARLS), where chronic pain independently predicted incident sarcopenia (HR 1.36, 95% CI 1.14–1.62), multi-site pain showed stepwise risk escalation (HR 1.79 for ≥3 sites), and high-sensitivity CRP (hsCRP) served as a significant mediator—providing prospective longitudinal evidence consistent with a temporally ordered relationship between pain-driven inflammation and sarcopenic progression, though residual confounding from shared risk factors cannot be excluded in this observational design [22]. A multinational cross-sectional analysis of 14,585 adults aged ≥65 years from six low- and middle-income countries (WHO SAGE cohort) further documented severe pain associated with 1.53-fold higher sarcopenia odds (adj. OR 1.53, *p* < 0.001), with a dose–response relationship between pain intensity and sarcopenia prevalence robust across age, sex, and country strata [23]. Quantification of the inflammatory substrate advanced through high-quality meta-analyses of observational data. Synthesis of 11,249 adults across 17 studies documented significantly elevated CRP (SMD 0.51, 95% CI 0.27–0.76) and IL-6 (SMD 0.24, 95% CI 0.03–0.45) in sarcopenic adults, alongside TNF-α trends—confirming population-level biomarker associations [24]. These findings were updated and expanded by meta-analysis of 3902 older adults across 21 studies, reporting IL-6 elevation (SMD 0.31, 95% CI 0.18–0.44), TNF-α elevation (SMD 0.24), and CRP elevation (SMD 0.36), with meta-regression confirming a dose–response relationship between cytokine load and sarcopenia severity [10].

#### 3.1.2. NF-κB–Driven Sarcopenic Muscle Catabolism

Experimental evidence from murine models is consistent with NF-κB serving as a molecular executor of inflammaging-driven sarcopenic wasting, though direct causal evidence in human sarcopenia remains to be established (Table 3). In a muscle-specific IKKβ-transgenic murine model, constitutive NF-κB activation drove MuRF-1 upregulation approximately 7-fold and induced MAFbx expression, resulting in fiber cross-sectional area reductions of 50% and total muscle mass losses of 30%, with elevated proteasomal flux [25]. Critically, both IKKβ pharmacological blockade and MuRF-1 knockout partially reversed this wasting phenotype, suggesting that NF-κB activation may be a key driver of sarcopenic-grade muscle loss through the ubiquitin–proteasome axis in this experimental model [25]. Prospective cohort analysis of 3075 community-dwelling adults aged ≥70 years (Health ABC cohort) confirmed population-level relevance, documenting IL-6 concentrations in the top tertile associated with a 1.65-fold higher risk of appendicular muscle mass loss over 2.5 years, TNF-α independently predicting grip-strength decline, and the highest IL-6 tertile conferring a 1.8-fold increased risk of disability [9]. The gut–muscle inflammatory axis constitutes an additional NF-κB activation pathway directly relevant to sarcopenic older adults. In a controlled in vivo experiment (Zhu et al., 2024; *n* = aged C57BL/6 mice), fecal microbiota transplantation from young donors reduced circulating LPS and restored intestinal barrier integrity, with concurrent attenuation of muscle TLR4/NF-κB signaling, downregulation of catabolic ubiquitin ligases MuRF-1 and Atrogin-1, and significant increases in grip strength and muscle cross-sectional area relative to aged-donor controls [28].

#### 3.1.3. NF-κB–Driven Nociceptive Sensitization

The same NF-κB–inflammatory circuit driving muscle catabolism simultaneously sensitizes nociceptive pathways at peripheral and central levels. In Swiss male mice, intramuscular and intraplantar IL-6 injection dose-dependently induced mechanical hyperalgesia lasting ≥4 h; this effect was fully blocked by anti-IL-6 receptor antibody, JAK2 inhibition, and NF-κB inhibition, with mechanistic association to TRPV1 and ASIC3 upregulation in dorsal root ganglion neurons—providing direct pharmacological evidence for IL-6/NF-κB–driven peripheral nociceptor sensitization [27]. At the central level, brief TNF-α exposure (15 min) in a murine spinal astrocyte model triggered sustained MCP-1/CCL2 release via JNK activation; intrathecal injection of TNF-α-activated astrocytes produced durable mechanical allodynia in naïve animals—an effect fully reversed by MCP-1 neutralizing antibody and MCP-1 siRNA—suggesting glial-to-neuron cytokine amplification as a plausible substrate of central sensitization [29]. Voxel-based morphometry in 26 chronic back pain patients versus matched controls documented 5–11% neocortical gray matter density reduction correlating with pain duration at 1.3 cm^3^/yr, with confirmed dorsolateral prefrontal cortex and thalamic reorganization—indicating that prolonged central sensitization was associated with structural remodeling of pain-inhibitory circuits in the included study population [26].

**Table 3:** Exercise-induced myokine signaling studies: molecular mechanisms in sarcopenia and chronic pain.

Study	Participants/Model	Study Design	Measures	Key Findings	Quality
Steensberg et al., 2000 [30]	6 healthy males; one-legged dynamic knee-extensor model	Controlled human experiment (a–fv balance)	Arterial & femoral-venous IL-6 (exercising vs. resting leg) Net IL-6 releases from contracting muscle 5-h exercise at 25 W (40% Wmax)	Arterial plasma IL-6 rose 19-fold vs. rest Net IL-6 releases from exercising leg 17-fold higher than arterial rise Resting leg showed no IL-6 release → contracting muscle is the direct source	NOS: 8/9
Keller et al., 2001 [31]	6 male subjects; two-legged dynamic knee-extensor (180 min)	Controlled human crossover (normal vs. low muscle glycogen)	IL-6 gene transcriptional activation (nuclear run-on) Muscle IL-6 mRNA abundance Plasma IL-6 kinetics	Low-glycogen trial: IL-6 transcription ↑ 40-fold at 90 min, 60-fold at 180 min Muscle IL-6 mRNA ↑ >100-fold (low glycogen) vs. 30-fold (control) Plasma IL-6 rise 16-fold vs. 10-fold (*p* < 0.05) → contractile/metabolic drive of IL-6	NOS: 8/9
Quinn et al., 2002 [32]	Mouse C2 skeletal myoblast/myotube line	In vitro experimental (retroviral IL-15 overexpression)	Sarcomeric myosin heavy chain (MHC), α-actin accumulation Myotube diameter/hypertrophic morphology Protein synthesis & degradation flux	IL-15 overexpression → MHC ↑ 5-fold; α-actin accumulation ↑ Hypertrophic morphology comparable to IGF-I-overexpressing myotubes IL-15 stimulated synthesis AND inhibited degradation → anti-atrophic myokine confirmed	NOS: 8/9
Wrann et al., 2013 [33]	C57BL/6 mice and Pgc1a^−/−^ mice; primary cortical neurons	Controlled in vivo (endurance running) + in vitro	Muscle & hippocampal FNDC5 expression PGC-1α-dependent FNDC5 regulation (Pgc1a^−/−^ brain) Hippocampal BDNF and neuroprotective gene induction	Endurance exercise ↑ FNDC5 in hippocampus via PGC-1α axis Forced FNDC5 expression ↑ Bdnf; FNDC5 knockdown ↓ Bdnf in neurons Peripheral FNDC5 delivery ↑ circulating irisin → Bdnf ↑ in hippocampus (muscle-brain crosstalk)	NOS: 9/9
Quinn et al., 2013 [34]	Male IL-15 transgenic mice vs. littermate controls	Controlled in vivo experiment (genetic model + run-to-exhaustion)	Endurance capacity (run-to-exhaustion) Substrate utilization (RER, fat oxidation) Fast-muscle PPARδ, SIRT1, PGC-1α/β expression	IL-15 Tg mice ran 2× longer than controls Preferential fat oxidation for energy metabolism Fast muscles: PPARδ, SIRT1, PGC-1α, PGC-1β ↑ → exercise-like oxidative phenotype	NOS: 8/9
Koltai/Radak et al., 2010 [35]	Young vs. old male Wistar rats; treadmill-trained	Controlled in vivo experiment (aging × exercise)	Skeletal-muscle SIRT1, SIRT6 level/activity NAD^+^ levels & NAMPT activity Redox markers (carbonylated proteins, HIF-1α, VEGF)	Exercise ↑ SIRT1 activity in aged rats via NAMPT-driven NAD^+^ biosynthesis Age-associated SIRT6 rise attenuated by training Exercise normalized redox imbalance → SIRT1-dependent anti-inflammaging mechanism	NOS: 8/9
Liu & Chang, 2018 [36]	Type-2 diabetic db/db mice vs. lean controls (±treadmill)	Controlled in vivo experiment (8-wk moderate exercise)	IκBα/NF-κB signaling & cytokine mRNA (IL-6, TNF-α, F4/80) SIRT1, AMPKα, PGC-1α activation; mitochondrial biogenesis (Nrf1, Tfam) MuRF-1, K48-polyubiquitination; TA & gastrocnemius CSA	Exercise ↓ NF-κB activation and ↓ IL-6/TNF-α mRNA in db/db muscle SIRT1-AMPK-PGC-1α axis and mitochondrial complex IV activity ↑ MuRF-1 ↓; TA CSA ↑ (830.6 vs. 676.5 µm^2^) → diabetic muscle atrophy prevented	NOS: 9/9
Hu et al., 2025 [37]	Pre-diabetic mellitus (PDM) mice; treadmill 12 m/min, 60 min/day × 5 d/wk × 4 wk	Controlled in vivo experiment + in vitro (high-glucose myocytes)	Skeletal-muscle FNDC5/irisin expression NLRP3 inflammasome, IL-1β, IL-18 Fasting glucose, insulin, lipid profile; muscle remodeling	Aerobic exercise ↑ FNDC5/irisin (*p* < 0.05) and ↓ NLRP3, IL-18 (*p* < 0.01) in PDM muscle Effect comparable to NLRP3 inhibitor MCC950 (pharmacologic control) Recombinant irisin blunted high-glucose–induced NLRP3/IL-1β/IL-18 → irisin-NLRP3 axis causal	NOS: 8/9
Gao et al., 2010 [29]	Adult C57BL/6 mice; cultured cortical & spinal astrocytes	Controlled in vivo + in vitro (intrathecal astrocyte injection)	Astrocyte MCP-1 expression/release (ELISA, IHC) JNK pathway activation Mechanical allodynia (von Frey) after i.t. astrocyte or TNF-α	Brief TNF-α (15 min) → sustained MCP-1 release from astrocytes via JNK Intrathecal TNF-α-activated astrocytes → mechanical allodynia in naïve mice Allodynia reversed by MCP-1 neutralizing antibody & MCP-1 siRNA → glia-to-neuron central sensitization	NOS: 9/9
Negaresh et al., 2019 [38]	31 elderly men 55–70 yrs (16 sarcopenic, 15 healthy)	Controlled human trial (8-wk whole-body progressive RT)	Serum myostatin & follistatin (ELISA) Quadriceps cross-sectional area 1RM squat & bench press; testosterone, IGF-1	Myostatin ↓ significantly in both sarcopenic & healthy elderly after RT (*p* < 0.05) Follistatin ↑ and QCSA ↑ (hypertrophy) post-training Strength gain greater in sarcopenic group → RT rescues myokine axis in sarcopenia	NOS: 8/9
Khalafi et al., 2023 [39]	768 adults (18–82 yrs) across 26 randomized studies (36 intervention arms)	Systematic review & meta-analysis	Circulating myostatin & follistatin (pre/post RT) Subgroup analyses: age, training duration, RT volume Heterogeneity (I^2^), risk of bias (Cochrane)	RT ↓ myostatin: SMD −1.31 (95% CI −1.74 to −0.88, *p* = 0.001; 26 studies) RT ↑ follistatin: SMD 2.04 (95% CI 1.51 to 2.52, *p* = 0.001; 14 studies) Effect robust across age and training duration → causal myokine adaptation to RT	AMSTAR-2: High

IL-6: interleukin-6; IL-15: interleukin-15; IL-1β: interleukin-1 beta; IL-18: interleukin-18; TNF-α: tumor necrosis factor alpha; MCP-1: monocyte chemoattractant protein-1 (CCL2); JNK: c-Jun N-terminal kinase; NF-κB: nuclear factor-kappa B; IκBα: inhibitor of kappa B alpha; NLRP3: NOD-like receptor pyrin domain-containing 3; FNDC5: fibronectin type III domain-containing 5 (irisin precursor); BDNF: brain-derived neurotrophic factor; PGC-1α/β: PPAR-γ coactivator-1 alpha/beta; PPARδ: peroxisome proliferator-activated receptor delta; SIRT1: sirtuin-1; SIRT6: sirtuin-6; NAD^+^: nicotinamide adenine dinucleotide; NAMPT: nicotinamide phosphoribosyltransferase; AMPKα: AMP-activated protein kinase α; MuRF-1: muscle RING-finger protein-1; MHC: myosin heavy chain; CSA: cross-sectional area; TA: tibialis anterior; RT: resistance training; 1RM: one-repetition maximum; QCSA: quadriceps cross-sectional area; RER: respiratory exchange ratio; PDM: pre-diabetic mellitus; MCC950: specific NLRP3 inflammasome inhibitor; a–fv: arterio–femoral-venous difference; Wmax: peak power output; i.t.: intrathecal; IGF-1: insulin-like growth factor-1; SMD: standardized mean difference; NOS: Newcastle–Ottawa Scale; AMSTAR-2: A Measurement Tool to Assess Systematic Reviews 2.

### 3.2. Exercise-Induced Myokine Signaling: Molecular Mechanisms

Eleven studies examined the molecular mechanisms of exercise-induced myokine signaling, comprising controlled human experiments, animal models, and systematic reviews with meta-analyses. Across the 12 studies examining myokine mechanisms, exercise-induced secretion of IL-6, IL-15, irisin, BDNF, and myostatin was consistently associated with suppression of NF-κB–driven inflammation, though the magnitude and direction of effects varied by exercise modality, intensity, and population characteristics.

#### 3.2.1. IL-6 and the AMPK–SIRT1–NF-κB Suppression Axis

In six healthy males, arteriovenous balance measurements during 5 h of one-legged dynamic knee-extensor exercise at 25 W (approximately 40% Wmax) demonstrated that arterial plasma IL-6 rose 19-fold, with net IL-6 release from the exercising limb 17-fold higher than arterial concentrations and no release from the resting contralateral leg—consistent with contracting muscle serving as a primary source of circulating IL-6 during exercise [30]. This response amplifies with metabolic substrate depletion: in a crossover experiment in six male subjects comparing glycogen-depleted versus normal muscle during 180 min two-legged knee-extensor exercise, IL-6 transcriptional activation reached approximately 40-fold at 90 min and approximately 60-fold at 180 min in the low-glycogen condition, with muscle IL-6 mRNA exceeding 100-fold induction versus approximately 30-fold in controls, and circulating IL-6 rising 16-fold versus 10-fold (*p* < 0.05)—consistent with a fuel-sensing transcriptional mechanism amplifying contractile IL-6 output in proportion to metabolic substrate availability [31]. Exercise suppresses NF-κB transcriptional activity in skeletal muscle through the SIRT1–AMPKα–PGC-1α axis, as characterized in type-2 diabetic db/db mice following 8 weeks of moderate-intensity treadmill exercise: SIRT1–AMPKα–PGC-1α activation, mitochondrial complex IV elevation, MuRF-1 and K48-polyubiquitination reduction, and tibialis anterior CSA restoration (830.6 vs. 676.5 μm^2^) were documented alongside significant NF-κB and pro-inflammatory cytokine attenuation [36]. The age-dependency of this pathway was demonstrated in young and old treadmill-trained Wistar rats, where exercise increased SIRT1 activity in aged muscle through NAMPT-driven NAD^+^ biosynthesis—consistent with SIRT1 upregulation as a key anti-inflammaging mechanism of regular exercise in aging skeletal muscle, at least in this murine model [35].

#### 3.2.2. IL-15 and Muscle Anabolic Signaling

IL-15, a myokine secreted in proportion to contraction volume, exerts direct anabolic effects on skeletal muscle through PI3K/Akt/mTOR signaling. Retroviral IL-15 overexpression in mouse C2 skeletal myotubes increased sarcomeric myosin heavy chain accumulation 5-fold and stimulated hypertrophic morphology comparable to IGF-I overexpression, while simultaneously enhancing protein synthesis and inhibiting degradation flux—suggesting IL-15 as a candidate anti-atrophic myokine with dual anabolic/anti-catabolic activity in this in vitro model, pending confirmation in human sarcopenic populations [32]. In vivo extension using IL-15 transgenic mice demonstrated approximately twice the running endurance of littermate controls at exhaustion, preferential fat oxidation as a fuel strategy, and significant upregulation of PPARδ, SIRT1, PGC-1α, and PGC-1β in fast-twitch muscles—consistent with systemic IL-15 elevation recapitulating an exercise-like oxidative phenotype in this transgenic murine model, suggesting potential mechanistic relevance to sarcopenia-associated metabolic impairments [34].

#### 3.2.3. Irisin and NLRP3 Inflammasome Suppression

Irisin, cleaved from FNDC5 during exercise through PGC-1α–dependent transcription, constitutes a mechanistically important myokine for both sarcopenia and pain outcomes through its capacity to suppress NLRP3 inflammasome activation. In C57BL/6 and Pgc1α^−/−^ mice, endurance exercise upregulated hippocampal FNDC5 expression via the PGC-1α axis; forced FNDC5 expression increased hippocampal BDNF while knockdown reduced it, and peripheral FNDC5 delivery elevated both circulating irisin and hippocampal BDNF—consistent with the FNDC5–irisin–BDNF axis as a muscle-to-brain signaling pathway that may modulate central pain-inhibitory capacity in this murine model [33]. In pre-diabetic mice undergoing 4-week aerobic exercise training, FNDC5/irisin significantly upregulated (*p* < 0.05) with concurrent reductions in NLRP3 inflammasome components and IL-18 (*p* < 0.01) in skeletal muscle; this effect matched pharmacological NLRP3 inhibitor MCC950 treatment, and recombinant irisin directly blunted high-glucose–induced NLRP3/IL-1β/IL-18 upregulation in vitro—consistent with an irisin–NLRP3 suppression axis in this pre-diabetic mouse model, suggesting exercise-induced irisin as a plausible upstream attenuator of NLRP3-driven inflammaging, though human evidence in sarcopenic older adults is currently absent [37].

#### 3.2.4. Myostatin Suppression and Anti-Nociceptive Effects

Myostatin, a TGF-β superfamily member that negatively regulates muscle mass, is consistently suppressed by resistance training across populations. In 31 elderly men aged 55–70 years (16 sarcopenic, 15 non-sarcopenic) undergoing 8-week progressive resistance training, serum myostatin significantly decreased in both groups (*p* < 0.05), with concurrent follistatin increases, quadriceps CSA gains by Magnetic Resonance Imaging, and one-repetition maximum (1RM) strength improvements—disproportionately greater in the sarcopenic group, indicating preferential rescue of the myostatin–follistatin axis in those with pronounced deficits [38]. Population-level robustness and magnitude emerged in a meta-analysis of 26 RCTs (*n* = 768, 36 intervention arms): resistance training reduced circulating myostatin by SMD −1.31 (95% CI −1.74 to −0.88, *p* = 0.001) and elevated follistatin by SMD 2.04 (95% CI 1.51 to 2.52, *p* = 0.001), with effects robust across age, training duration, and RT volume subgroups—suggesting myostatin suppression as a frequently observed and potentially clinically relevant adaptation to resistance training [39].

### 3.3. Clinical Outcomes: Exercise Modality Efficacy

Ten studies reported clinical outcomes of exercise modality efficacy for concurrent pain and sarcopenia management, including RCTs and systematic reviews with meta-analyses. Across the nine clinical studies, multicomponent training emerged as the modality most consistently associated with concurrent improvements in both pain and muscle outcomes, although direct evidence in populations with co-confirmed sarcopenia and chronic pain remains limited.

#### 3.3.1. Resistance Training

Progressive resistance training (PRT) produces the most consistently documented gains in muscle strength and functional capacity in older adults, with concurrent analgesic effects in musculoskeletal pain conditions (Table 4). A Cochrane systematic review and meta-analysis of 121 RCTs (*n* = 6700 community-dwelling and institutionalized older adults) reported PRT improved muscle strength (SMD 0.84, 95% CI 0.67–1.00), gait speed (MD +0.08 m/s), and Timed Up-and-Go performance (−4.3 s), while reducing osteoarthritis pain intensity (SMD −0.30) with a low adverse-event profile [40]. In older women with diagnosed sarcopenia (12 RCTs, *n* = 518), resistance training improved handgrip strength (SMD 0.43, 95% CI 0.11–0.74), gait speed (SMD 0.37, 95% CI 0.09–0.64), and knee-extension strength, with combined RT modalities yielding largest gains; skeletal muscle mass index changes remained non-significant, indicating RT targets strength and function over mass recovery in established sarcopenia [41]. For the dual knee osteoarthritis–sarcopenia phenotype, resistance exercise training (RET) combined with protein supplementation produced greater gait-speed recovery than RET alone (SMD 0.53, *p* < 0.01) and reduced WOMAC pain scores in older adults, with the combined intervention group demonstrating greater improvements than RET alone [42].

#### 3.3.2. Aerobic Exercise

Aerobic exercise training exerts primary benefit in this population through sustained suppression of circulating inflammaging biomarkers rather than direct muscle mass gains. Meta-analysis of 17 RCTs in older adults with chronic low-grade inflammation demonstrated that aerobic exercise reduced circulating IL-6 (MD −0.939 pg/mL, 95% CI −1.569 to −0.309, *p* = 0.004) and hsCRP (MD −0.853 mg/L, *p* < 0.05), with the largest effects in programs ≥12 weeks at moderate intensity—establishing a minimum three-month duration threshold for clinically meaningful inflammaging suppression [43]. In older adults with concurrent knee OA and sarcopenia, aerobic training alone produced non-significant appendicular lean mass gains (SMD not reported for aerobic-only arm), while combined RET with protein supplementation achieved significantly greater gait-speed recovery (SMD 0.53, *p* < 0.01) than RET alone—supporting multimodal combination as the preferred approach for concurrent dual-outcome benefit [42].

#### 3.3.3. Multicomponent Training

Multicomponent exercise programs—integrating resistance, aerobic, balance, and neuromotor elements—produce superior concurrent outcomes across both target conditions relative to single-modality protocols. A meta-analysis of 11 trials (*n* = 2222 cognitively frail older adults) demonstrated that multicomponent training significantly improved lower-limb muscle strength (MD +4.30, *p* < 0.001), grip strength (SMD 0.39, *p* = 0.008), frailty status (MD −2.21, *p* < 0.001), and depression scores (MD −1.20, *p* = 0.001), with programs exceeding 120 min/week incorporating both aerobic and resistance components yielding greatest benefit [44].

International consensus synthesis across the ICFSR evidence base identified progressive resistance training (2–3 times/week at 70–80% 1RM) as sufficient for meaningful gains in strength, power, and hypertrophy, while designating multicomponent training (RT + aerobic + balance) as the superior prescription for concurrent sarcopenia and frailty management—providing evidence for the integrated prescription framework described in Section 4.4 [45]. A key practical barrier to multicomponent training adoption remains pain-related kinesiophobia and fear-avoidance behavior. Clinical evidence supports a two-phase prescription architecture: PNE in older adults with chronic pain significantly reduced kinesiophobia (TSK-11) and pain-related disability while improving gait speed [48]; a 12-week quasi-RCT of PNE combined with physical activity in 42 older women with chronic low-back pain produced large-effect improvements in chair-stand performance (d = 0.88) and daily step count (d = 0.87), with concurrent reductions in pain catastrophizing (d = −0.87) and fear-avoidance beliefs (d = −0.65) [49]. This sequencing carries direct mechanistic rationale for optimizing myokine responses in older adults with concurrent chronic pain and sarcopenia, though it remains unreflected in current international sarcopenia exercise guidelines.

## 4. Discussion

The evidence synthesized in this review is consistent with a unified pathophysiological model in which inflammaging operates as a plausible shared mechanistic contributor to both chronic pain and sarcopenia in aging populations, simultaneously sustaining nociceptive sensitization and promoting progressive muscle catabolism across included studies. Interventions that suppress inflammaging may therefore simultaneously reduce pain burden and attenuate sarcopenic progression through this hypothesized shared pathway—a therapeutic possibility that single-disease pharmacological approaches have not addressed, and one that awaits direct empirical validation in adequately powered trials using dual eligibility criteria.

### 4.1. Inflammation as a Causal Intermediary in the Pain–Sarcopenia Cycle

The conventional clinical model of the pain–sarcopenia relationship assigns primacy to physical inactivity: pain limits movement, and reduced movement accelerates muscle loss [5,6]. The longitudinal data reviewed here are inconsistent with this as a complete explanation. The dose–response gradient between anatomical pain distribution and incident sarcopenia risk—HR 1.79 for ≥3 pain sites versus HR 1.36 for single-site pain—cannot be attributed to a proportional increase in movement restriction across pain sites, because the degree of physical limitation in chronic musculoskeletal pain does not distribute anatomically in a manner that would produce this gradient [22]. Wider anatomical spread of peripheral sensitization plausibly sustains a proportionally larger afferent-driven systemic cytokine output [6,27], maintaining the NF-κB transcriptional environment in skeletal muscle that drives MuRF-1 and MAFbx upregulation [6,25]. The partial mediation of this relationship by hsCRP in the CHARLS prospective dataset provides the strongest available human evidence for this interpretation [50]. Its implication is direct: pain management strategies that attenuate nociceptive signaling without concurrently suppressing systemic inflammatory tone—including opioids, NSAIDs, and TNF-α inhibitors—will fail to interrupt sarcopenic progression at its mechanistic root, regardless of secondary improvements in physical activity [4,11,12].

The structural neuroimaging evidence provides an additional mechanistic dimension that warrants integration into musculoskeletal pain and sarcopenia research [26,51]. Gray matter attrition in the dorsolateral prefrontal cortex and thalamus at 1.3 cm^3^/yr is not a passive correlate of pain duration; these structures are the primary substrates of descending noradrenergic and serotonergic inhibitory projections to the dorsal horn, and their atrophy degrades conditioned pain modulation efficiency in proportion to duration [26]. The reduced exercise-induced hypoalgesia (EIH) in chronic pain populations is mechanistically downstream of exactly this structural erosion [46]. In sarcopenic older adults with longstanding chronic pain, the analgesic ceiling of any exercise intervention is therefore lower at baseline than that in pain-free counterparts—not because of inadequate exercise prescription, but because the central inhibitory substrate through which EIH operates has been progressively degraded [46,52]. Achieving clinically meaningful pain reduction through exercise in this population requires a myokine secretory stimulus sufficient not merely to produce transient analgesia but to support the structural and functional recovery of prefrontal inhibitory circuits over time—a threshold quantitatively higher than what is required in populations without central sensitization [33,53].

### 4.2. Exercise Dose Thresholds for Myokine Output

The SIRT1/AMPK/NF-κB mechanistic data establish a specific problem for translational exercise prescription: SIRT1-mediated NF-κB deacetylation is stoichiometrically dependent on NAD^+^ availability, which is suppressed in sarcopenic aging muscle through SASP-driven poly-ADP-ribose polymerase-1 hyperactivation [54,55]. In older adults with active inflammaging, the SIRT1 activity available at rest is already operating below the deacetylation threshold for NF-κB p65—meaning that only exercise stimuli generating sufficient AMPK activation to drive NAMPT-dependent NAD^+^ biosynthesis above this suppressed baseline will produce net NF-κB attenuation [35,56,57]. This interpretation is supported by experimental data showing concurrent attenuation of NF-κB–driven inflammation and muscle atrophy in exercised versus sedentary diabetic models, an effect consistent with SIRT1/AMPK axis activation as the primary mediating mechanism. The CSA recovery observed specifically in moderately exercised—not low-intensity exercised—db/db mice is the experimental correlate of this threshold [36]. Sub-threshold exercise stimuli do not simply produce smaller SIRT1 effects; in an NAD^+^-depleted cellular environment, they may produce no effective NF-κB deacetylation [36,58].

The glycogen depletion data compound this dose-dependency argument. The >100-fold amplification of muscle IL-6 transcription under low-glycogen conditions versus 30-fold under euglycemic conditions reflects a calmodulin-dependent protein kinase II-dependent, NF-κB-independent transcriptional mechanism in which intramuscular fuel status is the primary upstream determinant of paracrine anti-inflammatory IL-6 output [31,59,60]. This dose–response relationship implies that in sarcopenic older adults with impaired glycogen resynthesis, the anti-inflammatory IL-6 secretory response to exercise will be blunted unless training sessions achieve sufficient metabolic loading to substantially deplete intramuscular glycogen—a stimulus that exercise at below approximately 60% VO_2_max for less than 30 min does not reliably generate in aging muscle [61,62]. The elastic band programs, aquatic sessions, and low-load resistance protocols that dominate chronic pain exercise prescriptions in older adults fall below both the AMPK activation threshold and the glycogen depletion threshold identified here as prerequisites for therapeutically meaningful myokine output [63].

Myostatin suppression through mechanical loading represents an additional mechanically driven pathway relevant to muscle hypertrophy in this context. Resistance training inhibits myostatin activity, thereby relieving inhibition of Akt signaling and permitting mTORC1-dependent activation of downstream hypertrophic pathways [64,65]. Mechanical stress from resistance exercise also activates ERK1/2 and Akt pathways, mediating muscle protein synthesis through pathways independent of endurance-driven adaptations [66,67,68]. The myostatin–follistatin axis responds reliably to mechanical loading, with resistance exercise upregulating follistatin, neutralizing myostatin, and activating muscle satellite cells [69]. Clinical data suggest that progressive resistance training was associated with suppression of age-related myostatin elevation and attenuation of sarcopenic progression in older adults [69,70].

Consequently, optimizing muscle adaptation requires training stimuli that simultaneously target distinct metabolic and mechanical thresholds. Moderate-to-high-intensity exercise not only attenuates muscle protein degradation by activating the SIRT1/AMPK axis to suppress NF-κB signaling, but also provides sufficient mechanical tension to stimulate follistatin expression and Akt-mediated protein synthesis [36,69,71].

Moderate to high-intensity exercise attenuates muscle protein degradation by activating the SIRT1/AMPK axis to suppress NF-κB signaling and simultaneously provides sufficient mechanical tension to stimulate follistatin expression and Akt-mediated protein synthesis [36,71]. This intersection of metabolic (SIRT1/AMPK) and mechanical (myostatin/Akt) signaling underscores the necessity of comprehensive resistance protocols in this population [69,71].

### 4.3. The Gut–Muscle–Pain Axis

Recent evidence indicates that gut microbiota dysbiosis serves as a shared upstream modulator of both skeletal muscle atrophy and central pain sensitization [72,73,74,75,76]. Impaired intestinal barrier function allows gut-derived LPS to translocate into the systemic circulation, establishing metabolic endotoxemia [77]. In skeletal muscle, systemic LPS binds to TLR4 and activates the NF-κB signaling pathway, which directly upregulates the expression of muscle-specific E3 ubiquitin ligases, MuRF-1 and Atrogin-1, driving sarcopenic catabolism [73,74,78]. Emerging evidence indicates that targeted gut microbiota modulation via probiotics or prebiotics, independent of exercise, suppresses the LPS–TLR4–NF-κB signaling axis and reduces MuRF-1 and Atrogin-1 expression, attenuating muscle atrophy in preclinical models [73,74,78]. Mechanistically, this same gut-derived LPS simultaneously impacts the nervous system by activating TLR4 expressed on spinal microglia and dorsal root ganglion macrophages [75,79]. This microglial and macrophage activation shifts toward a pro-inflammatory phenotype, releasing neuroinflammatory cytokines (TNF-α, IL-1β) that directly depolarize spinal nociceptive neurons and sustain central sensitization [76,80]. The LPS–TLR4 pathway thus operates as a shared upstream node linking intestinal dysbiosis to sarcopenic catabolism and chronic neuropathic pain simultaneously, reinforcing the inadequacy of single-condition treatment frameworks for this comorbid population. Translational data from FMT experiments in aged murine models further suggest that the gut-derived LPS–TLR4–NF-κB axis represents a potentially modifiable upstream driver of sarcopenic catabolism, warranting direct investigation in human populations with comorbid sarcopenia and chronic pain.

These threshold dependencies carry direct implications for exercise prescription, as moderate-intensity aerobic training at ≥150 min/week consistently increases the relative abundance of short-chain fatty acid-producing taxa while reducing LPS-producing Gram-negative populations in older adults [81,82]. The anti-inflammatory effects of this microbial shift—reduced circulating LPS, attenuated TLR4/NLRP3 activation, lower basal NF-κB drive in both muscle and spinal cord—operate in parallel with and are additive to the direct myokine-secretory effects of the same training stimulus [77,83]. Accordingly, the minimum aerobic volume required to produce clinically meaningful anti-inflammaging effects is likely determined not by myokine secretory thresholds alone but by the combined threshold for both myokine output and gut microbiome remodeling—meaning that prescriptions falling below this combined threshold may be less likely to achieve both myokine-secretory and microbiome-remodeling effects simultaneously. Nonetheless, low-intensity exercise that is insufficient to engage SIRT1/AMPK-dependent myokine thresholds may still produce clinically meaningful gut microbiota shifts and partial TLR4–NF-κB attenuation, making it a valuable starting point for frail or highly deconditioned older adults who cannot initially achieve higher training intensities. Beyond myokine signaling, the broader metabolic context of inflammaging warrants consideration. Serum 25-hydroxyvitamin D deficiency is independently associated with osteoarthritis progression and functional decline in older adults [84], and vitamin D signaling attenuates NF-κB–driven pro-inflammatory cytokine production—suggesting a mechanistic intersection with the myokine axes reviewed here. Future frameworks should therefore consider integrating vitamin D status alongside myokine profiling as complementary biomarkers of the shared inflammaging substrate.

### 4.4. A Two-Phase Prescription Framework

The clinical evidence reviewed in Section 3.3 suggests that training volume and component breadth, rather than modality alone, may be key determinants of concurrent outcomes in frail and sarcopenic older adults, providing the mechanistic rationale for the integrated prescription framework described below. The ICFSR consensus [45]—progressive resistance training at 70–80% 1RM, 2–3 times/week, with multicomponent training for concurrent frailty and sarcopenia management—is consistent with the ERK/Akt mechanical threshold and, at ≥12 weeks, the SIRT1/AMPK duration threshold proposed in this review, though direct threshold validation in sarcopenic older adults with chronic pain has not been performed [41,85]. Meta-analytic evidence confirms that resistance training at these parameters produces meaningful gains in handgrip strength (SMD 0.43, 95% CI 0.11–0.74) and gait speed (SMD 0.37, 95% CI 0.09–0.64) in sarcopenic older women [41], with ≥12-week resistance training reducing circulating IL-6 by WMD −0.91 pg/mL and TNF-α by WMD −1.64 pg/mL in 278 sarcopenic Asian older adults across 6 RCTs [47]. For the dual knee OA–sarcopenia phenotype, the superior outcomes of combined resistance training and protein supplementation over exercise alone further support the rationale for multimodal intervention strategies in this population. Its limitation in the target population of this review is not mechanistic but behavioral: it was designed and validated in populations without concurrent chronic pain, and it contains no provision for the kinesiophobia and fear-avoidance behavior that systematically prevents older adults with chronic pain from reaching and maintaining the loading intensities at which this prescription is effective [86,87].

Fear-avoidance behavior is not a compliance problem amenable to motivational intervention; it is mechanistically rooted in prefrontal threat-appraisal processes that suppress voluntary motor output [88]. The elevated pain catastrophizing and kinesiophobia characteristic of centrally sensitized older adults suppress voluntary motor cortex output through prefrontal threat-appraisal mechanisms, functionally reducing the maximum voluntary contraction available during resistance training sessions independent of actual musculoskeletal capacity [88,89]. A participant whose pain catastrophizing maintains perceived threat during loading at 60% 1RM will not voluntarily escalate to 70–80% 1RM regardless of how progressive the prescription is written. The functional consequence is that kinesiophobia, left unaddressed, enforces a sub-threshold loading ceiling that prevents the ERK/Akt and SIRT1/AMPK activation thresholds identified in this review from being reached [90,91]. PNE in older adults with chronic pain significantly reduced kinesiophobia scores alongside meaningful improvements in gait speed and pain disability [49]. PNE combined with physical activity produced large-effect improvements in chair-stand performance (d = 0.88), daily step count (d = 0.87), pain catastrophizing (d = −0.87), and fear-avoidance beliefs (d = −0.65) in older women with chronic low-back pain [49]. The magnitude of these effects—achieved without pharmacological intervention or structured progressive resistance training—indicates that removal of the central threat-appraisal barrier to loading is itself a major determinant of whether exercise-based interventions generate the mechanical and metabolic stimuli required for myokine-driven dual-outcome benefit.

These data provide the mechanistic rationale for a hypothesis-driven, two-phase prescription proposal. The specific parameters that follow—the 4-week Phase 1 duration, the 70–80% 1RM resistance target, and the 65% VO_2_max aerobic target—are each derived from separate independent studies rather than from a single trial testing the combined framework as a whole, and therefore represent a synthesis-based proposal requiring prospective validation before clinical adoption. Phase 1 (weeks 1–4; adjustable to weeks 1–6 for more functionally limited individuals) delivers PNE as the primary intervention, targeting Tampa Scale for Kinesiophobia and PCS score reduction below the threshold at which fear-avoidance behavior attenuates voluntary motor drive; the explicit reconceptualization of exercise-induced IL-6 as a muscle-derived anti-inflammatory signal—rather than a pathological pro-inflammatory marker—is a mechanistically grounded component of this educational content for this population. This sequencing is not merely pedagogical but mechanistically necessary: central sensitization suppresses voluntary motor drive through fear-avoidance behavior, preventing older adults from reaching the mechanical loading threshold (70–80% 1RM) required to engage SIRT1/AMPK-dependent myokine secretion. Without first attenuating central sensitization through PNE, prescribed exercise intensities are likely to remain sub-threshold for meaningful anti-inflammatory myokine output regardless of prescription quality. Phase 2 (weeks 5 onward; weeks 7 onward for functionally limited subgroups) introduces progressive multicomponent training, with a suggested initial target of 40–60% 1RM for frail or functionally limited older adults, escalating toward 70–80% 1RM as tolerated for those with adequate baseline functional capacity, and 65% VO_2_max for aerobic components, maintained for a minimum of 12 weeks to approach the duration thresholds associated with clinically meaningful NF-κB suppression in the included evidence. This hypothesis-driven, two-phase PNE-facilitated proposal is not reflected in current ICFSR, EWGSOP2, or major chronic pain management guidelines. Its translation into clinical guidance is contingent on prospective validation in trials that enroll older adults with co-confirmed sarcopenia and chronic pain, test the integrated sequence as a whole rather than its components in isolation, and include subgroup analyses to determine whether intensity parameters require adjustment for frail or older individuals.

### 4.5. The Irisin–BDNF Axis as a Dual-Outcome Target

Irisin and BDNF occupy a distinct position among the myokines reviewed because their co-secretion through the PGC-1α/FNDC5 axis during exercise simultaneously addresses three pathophysiological domains that are otherwise targeted by separate mechanistic pathways: skeletal muscle anabolism, NLRP3 inflammasome suppression, and structural maintenance of central pain inhibitory circuits [92,93,94,95].

Irisin-mediated NLRP3 suppression achieves quantitative equivalence with the pharmacological inhibitor MCC950 at exercise-induced concentrations, suggesting NLRP3 as a plausible convergence point for SASP, mitochondrial reactive oxygen species, and LPS-TLR4-derived inflammaging inputs that irisin may simultaneously attenuate [37]. Irisin-mediated NLRP3 suppression would therefore attenuate IL-1β and IL-18 production driven by all three upstream pathways in parallel—cytokines that both directly sensitize peripheral nociceptors through TRPV1 modulation and sustain spinal microglial activation [96,97]. Concurrently, FNDC5-driven hippocampal BDNF elevation supports prefrontal and anterior cingulate structural integrity, whose progressive atrophy was quantified in chronic pain patients and whose preservation is required for sustained descending pain-inhibitory function [98,99]. Exercise-induced PGC-1α/FNDC5 activation drives concurrent irisin and BDNF secretion [100,101]; irisin-mediated NLRP3 suppression attenuates peripheral and spinal nociceptive sensitization [101]; and BDNF-supported prefrontal circuit integrity maintains descending inhibitory capacity [92,100]. Together, these mechanistically distinct but co-activated axes provide a plausible molecular basis for the attenuation of central sensitization in sarcopenic older adults with chronic pain—a hypothesis that remains to be tested in prospective trials.

Future studies co-measuring FNDC5/irisin and BDNF alongside NLRP3-pathway biomarkers (IL-1β, IL-18), conditioned pain modulation efficiency, and dual sarcopenia/pain criteria at baseline and post-intervention would provide the mechanistic resolution required to determine whether this axis mediates the concurrent clinical benefits predicted by this review.

### 4.6. Limitations and Future Directions

The mechanistic evidence base for the pathway proposed in this review is built substantially on murine and in vitro models, which establish causality but carry well-recognized limitations in translating to sarcopenic older adults with polypharmacy and multimorbidity. The inclusion of animal and in vitro studies without formal preclinical risk-of-bias appraisal (e.g., SYRCLE) introduces translational uncertainty that no post hoc appraisal strategy can entirely eliminate. No included trial enrolled older adults with co-confirmed sarcopenia and chronic pain as dual eligibility criteria while simultaneously measuring myokine profiles alongside both outcome domains; the integrated pathway from PNE-facilitated exercise prescription to concurrent dual-outcome benefit therefore remains an evidence-based mechanistic hypothesis rather than a directly demonstrated clinical effect. The gut–muscle–pain axis evidence is currently limited to animal FMT models and cross-sectional human microbiome data, with no direct evidence that exercise-induced gut microbiome remodeling attenuates LPS-driven NF-κB activation in human skeletal muscle. EIH data are predominantly from single acute exercise sessions, leaving the chronic adaptation trajectory of central inhibitory circuits under progressive training unstudied in populations with concurrent sarcopenia and pain. Additionally, heterogeneity in the timing of myokine blood sampling across included studies—ranging from acute post-exercise measurements to post-program chronic adaptation assessments—precluded direct cross-study comparison of myokine response magnitudes and limited the precision of dose–response inference. PNE trials were conducted without co-confirmed sarcopenia as an eligibility criterion, limiting inference about whether kinesiophobia reduction translates to the loading intensities required for anti-sarcopenic myokine output.

These limitations define trial design priorities. Future RCTs should enroll adults aged ≥65 years meeting both EWGSOP2/AWGS 2019 sarcopenia criteria and validated chronic pain criteria (numerical rating scale ≥ 4 for ≥3 months at ≥2 anatomical sites), randomize to PNE-facilitated multicomponent training versus standard-intensity exercise versus control, and incorporate standardized myokine profiling (IL-6, IL-15, irisin, BDNF, myostatin) at baseline and at 4, 12, and 24 weeks alongside CPM testing, gut microbiome profiling, and dual sarcopenia/pain outcome assessment—to provide the first direct test of the mechanistic convergence model advanced in this review.

## 5. Conclusions

Chronic musculoskeletal pain and sarcopenia share a unified molecular basis in inflammaging-driven NF-κB hyperactivation that simultaneously sustains sarcopenic catabolism and nociceptive sensitization, undermining the rationale for single-target pharmacological management and positioning structured exercise as a mechanistically justified therapeutic candidate. Exercise-induced myokines—including IL-6, IL-15, irisin, and BDNF—were associated with suppression of NF-κB transcriptional activity through AMPK/SIRT1-dependent pathways, alongside attenuation of central sensitization and restoration of anabolic capacity, as suggested by the included evidence. Among the exercise modalities evaluated, multicomponent training was most consistently associated with superior concurrent outcomes across both sarcopenia and pain domains in the included studies. These conclusions must be interpreted within the context of key methodological constraints: the heterogeneity of included study designs precluded meta-analytic pooling; the protocol was not prospectively registered in PROSPERO prior to data extraction, which represents a meaningful methodological limitation of this review. The absence of prospective registration increases the theoretical risk of selective outcome reporting and limits the verifiability of a priori decision-making. We acknowledge this constraint explicitly and recommend that future systematic reviews on myokine signaling in sarcopenia-associated chronic pain adopt PROSPERO registration prior to data collection. The incorporation of animal and in vitro models introduces translational uncertainty; most myokine dose–response data derive from pain-free rather than sarcopenic pain populations, and the predominantly European and East Asian cohort composition constrains generalizability. Most critically, no existing randomized controlled trial has enrolled older adults with co-confirmed sarcopenia and chronic pain as dual eligibility criteria, leaving the mechanistic convergence model advanced here empirically unvalidated. Future trials addressing this gap—incorporating standardized myokine profiling, conditioned pain modulation assessment, and strategies to overcome kinesiophobia-driven sub-threshold loading—are required to provide the first direct clinical test of the hypothesized integrated myokine–NF-κB–dual-outcome pathway proposed in this review.

## Figures and Tables

**Table 1:** Full search strategies used for each database.

Database	Search Query String
PubMed	(“inflammaging” OR “chronic inflammation” OR “low-grade inflammation”) AND (“sarcopenia” OR “muscle wasting”) AND (“chronic pain” OR “musculoskeletal pain”) AND (“exercise” OR “physical activity”) AND (“myokines” OR “IL-6” OR “irisin” OR “IL-15” OR “BDNF”)
Web of Science	(“inflammaging” OR “chronic low-grade inflammation”) AND (“sarcopenia” OR “muscle atrophy”) AND (“chronic pain” OR “nociception”) AND (“exercise” OR “resistance training”) AND (“NF-κB” OR “myokines” OR “cytokines”)
Scopus	(“inflammaging” OR “chronic inflammation”) AND (“sarcopenia”) AND (“chronic pain”) AND (“exercise” OR “physical training”) AND (“myokines” OR “irisin” OR “IL-15”)
Embase	(“inflammaging” OR “chronic low-grade inflammation” OR “senescence-associated secretory phenotype”) AND (“sarcopenia” OR “muscle wasting” OR “muscle atrophy”) AND (“chronic pain” OR “musculoskeletal pain” OR “nociception”) AND (“exercise” OR “resistance training” OR “physical activity”) AND (“myokines” OR “IL-6” OR “irisin” OR “BDNF” OR “NF-kB”)

**Table 2:** Key studies on inflammaging pathophysiology linking chronic musculoskeletal pain and sarcopenia.

Study	Participants/Model	Study Design	Measures	Key Findings	Quality
Visser et al., 2002 [9]	3075 community-dwelling adults ≥70 yrs (Health ABC cohort)	Prospective cohort (experimental/observational)	IL-6, TNF-α (plasma) Appendicular muscle mass (DEXA) Grip strength, lower-extremity performance	IL-6 > 1.80 pg/mL → lower muscle mass and lower muscle strength TNF-α > 3.20 pg/mL ↑ independently predicted grip-strength decline Highest IL-6 tertile: disability risk ↑	Low Risk
Cai et al., 2004 [25]	MIKK (muscle-specific IKKβ-transgenic) C57BL/6 mice; C2C12 myotubes; LPS/TNF-α challenge	In vivo + in vitro experimental	NF-κB activity (EMSA, luciferase) MuRF-1, MAFbx (qPCR) Fiber CSA Muscle wet mass Proteasomal flux	Muscle-specific NF-κB activation → MuRF-1 ↑ 7-fold Severe fiber atrophy (CSA ↓ 50%); total muscle mass ↓ 30% IKKβ blockade/MuRF-1 KO reversed wasting → causal role confirmed	NOS: 8/9
Apkarian et al., 2004 [26]	26 chronic back-pain patients (17 analyzed by VBM) vs. matched healthy controls	Cross-sectional neuroimaging (voxel-based morphometry)	Whole-brain gray-matter density (VBM) Pain duration (yrs) Cognitive-emotional pain processing	Neocortical gray-matter density ↓ 5–11% in CBP patients Gray-matter loss correlated with pain duration (1.3 cm^3^/yr loss) Dorsolateral prefrontal & thalamic structural reorganization confirmed	NOS: 8/9
Manjavachi et al., 2010 [27]	Swiss male mice; intramuscular/intraplantar IL-6 injection	Controlled animal experiment	Mechanical & thermal hyperalgesia (von Frey, hot-plate) TRPV1, ASIC3 expression (DRG) IL-6R/gp130/JAK2/NF-κB signaling	IL-6 dose-dependently induced muscle mechanical hyperalgesia (≥4 h) Hyperalgesia blocked by anti-IL-6R, JAK2 & NF-κB inhibitors → direct nociceptor sensitization TRPV1/ASIC3 upregulation in DRG neurons confirmed	NOS: 8/9
Bano et al., 2017 [24]	11,249 adults across 17 observational studies	Systematic review & meta-analysis	Serum CRP, IL-6, TNF-α Sarcopenia/muscle-mass status Heterogeneity and publication bias	CRP ↑ significantly in sarcopenic adults (SMD 0.51, 95% CI 0.27–0.76) IL-6 ↑ (SMD 0.24, 95% CI 0.03–0.45); TNF-α trend ↑ Inflammaging biomarkers quantitatively confirmed as sarcopenia correlates	AMSTAR-2: High
Chen et al., 2023 [4]	33,600 community-dwelling adults ≥60 yrs across 17 studies	Systematic review & meta-analysis	Chronic musculoskeletal pain prevalence Sarcopenia (EWGSOP/AWGS criteria)	Chronic pain → sarcopenia pooled OR 1.52 (95% CI 1.31–1.76) Bidirectional association confirmed (sarcopenia → pain OR 1.73 95% CI 1.54–1.95)	AMSTAR-2: High
Veronese et al., 2023 [21]	4102 community-dwelling adults (Health ABC cohort; 10-yr follow-up)	Prospective longitudinal cohort	Baseline self-reported pain (severity, interference) Incident sarcopenia (low lean mass + low strength) Inflammatory serum biomarkers	Moderate–severe pain: sarcopenia incidence HR 1.47 (95% CI 1.15–1.88) Pain-interference ↑ dose-dependently raised sarcopenia risk Association partially mediated by IL-6/CRP elevation	NOS: 8/9
Smith et al., 2023 [23]	14,585 adults ≥65 yrs from 6 LMICs (WHO SAGE)	Cross-sectional multinational epidemiological study	Chronic pain (severe pain in past 30 days) Sarcopenia (low calf-circumference proxy + slow gait) Socio-demographic/inflammatory covariates	Severe pain → 1.53-fold higher sarcopenia odds (adj. OR 1.53, *p* < 0.001) Dose–response: pain intensity ↑ sarcopenia prevalence ↑ Association robust across age, sex, country strata	NOS: 8/9
Ding et al., 2024 [10]	3902 older adults across 21 studies	Systematic review & meta-analysis	Circulating IL-6, TNF-α, CRP, IL-1β Sarcopenia status (EWGSOP2/AWGS) Heterogeneity (I^2^), sensitivity & meta-regression	IL-6 ↑ in sarcopenia (SMD 0.31, 95% CI 0.18–0.44) TNF-α ↑ (SMD 0.24) and CRP ↑ (SMD 0.36) Dose-response between cytokine load and sarcopenia severity confirmed	AMSTAR-2: High
Chen et al., 2024 [22]	5568 adults ≥45 yrs (China Health & Retirement Longitudinal Study)	Prospective longitudinal cohort (4-yr follow-up)	Baseline chronic pain (sites, severity) Incident sarcopenia (AWGS 2019 criteria) hsCRP and functional performance	Chronic pain → incident sarcopenia HR 1.36 (95% CI 1.14–1.62) Multi-site pain: stepwise ↑ sarcopenia risk (HR 1.79 for ≥3 sites) Pain-sarcopenia link mediated by elevated hsCRP	NOS: 9/9
Zhu et al., 2024 [28]	Aged C57BL/6 mice receiving fecal microbiota transplantation from young vs. aged donors	Controlled in vivo experiment (FMT)	Gut microbial composition (16S) Serum LPS; intestinal barrier markers Muscle TLR4/NF-κB; muscle mass & grip strength	Young-donor FMT: serum LPS ↓ and gut-barrier integrity restored Muscle TLR4/NF-κB signaling ↓; MuRF-1/Atrogin-1 ↓ Grip strength ↑ and muscle CSA ↑ vs. aged-donor FMT controls	NOS: 8/9

CSA: cross-sectional area; hsCRP: high-sensitivity C-reactive protein; CRP: C-reactive protein; DEXA: dual-energy X-ray absorptiometry; LPS: lipopolysaccharide; TLR4: Toll-like receptor 4; NF-κB: nuclear factor-kappa B; MuRF-1: muscle RING-finger protein-1; MAFbx: muscle atrophy F-box; IKKβ: inhibitor of κB kinase β; TRPV1: transient receptor potential vanilloid 1; ASIC3: acid-sensing ion channel 3; DRG: dorsal root ganglion; VBM: voxel-based morphometry; CBP: chronic back pain; EWGSOP: European Working Group on Sarcopenia in Older People; AWGS: Asian Working Group for Sarcopenia; FMT: fecal microbiota transplantation; SMD: standardized mean difference; OR: odds ratio; HR: hazard ratio; Health ABC: Health, Aging, and Body Composition study; LMIC: low- and middle-income country; NOS: Newcastle–Ottawa Scale; AMSTAR-2: A Measurement Tool to Assess Systematic Reviews 2.

**Table 4:** Clinical evidence on exercise modality efficacy for concurrent chronic pain and sarcopenia outcomes in older adults.

Study	Participants/Model	Study Design	Measures	Key Findings	Quality
Liu & Latham, 2009 [40]	6700 community-dwelling and institutionalized older adults (121 RCTs); mean age ≥60 yrs	Systematic review & meta-analysis (Cochrane) of progressive resistance training (PRT)	Muscle strength (1RM) and muscle power Physical function (gait speed, chair-stand, stair-climb, TUG) Disability, pain, and adverse events	PRT improved muscle strength: SMD 0.84 (95% CI 0.67–1.00; 33 studies) Gait speed ↑ (MD 0.08 m/s) and TUG ↓ 4.3 s; chair-stand and stair-climb improved significantly Reduced pain from OA (SMD −0.30) with low adverse-event rate → PRT benefits strength, function, and OA pain	AMSTAR-2: High
Liao et al., 2023 [42]	Older adults with concurrent knee OA and sarcopenia (systematic review & meta-analysis of RCTs)	Meta-analysis of resistance exercise training (RET) ± protein supplementation	Walking speed (gait-speed recovery) Muscle mass (ASM/SMI) and strength (1RM, knee-extension) Pain (WOMAC, VAS) and physical function	RET + protein supplementation > RET alone for gait-speed recovery (SMD 0.53, *p* < 0.01) RET ↑ knee-extension strength and ASM in KOA-sarcopenia cohorts WOMAC pain ↓ with RET; combined intervention most effective for dual KOA-sarcopenia phenotype	AMSTAR-2: High
Tayebi et al., 2025 [43]	17 RCTs; older adults (≈60–85 yrs) with chronic low-grade inflammation	Systematic review & meta-analysis of aerobic exercise	Circulating IL-6 and hsCRP (pre/post aerobic training) Subgroup analyses: age, training duration, intensity Heterogeneity (I^2^), risk of bias	Aerobic exercise ↓ IL-6: MD −0.939 pg/mL (95% CI −1.569 to −0.309, *p* = 0.004) Aerobic exercise ↓ hsCRP: MD −0.853 mg/L (*p* < 0.05) Greater effect with ≥12 wks moderate-intensity training → aerobic training suppresses inflammaging	AMSTAR-2: High
Zhou et al., 2025 [41]	12 RCTs; 518 older women with diagnosed sarcopenia	Systematic review & meta-analysis of resistance training (RT)	Handgrip strength, knee-extension strength, gait speed Skeletal muscle mass index (SMI) Subgroup analyses by RT modality (traditional, combined, elastic-band)	RT ↑ handgrip strength: SMD 0.43 (95% CI 0.11–0.74) RT ↑ gait speed: SMD 0.37 (0.09–0.64) and knee-extension strength Combined RT modalities most effective; SMI gains non-significant → RT targets strength & function	AMSTAR-2: High
Luo et al., 2024 [44]	11 trials; 2222 cognitively frail older adults (≥65 yrs)	Systematic review & meta-analysis of multicomponent exercise (aerobic + RT + balance)	Lower-limb muscle strength, grip strength Frailty status (Fried phenotype) Cognitive function and depression	Lower-limb strength ↑ MD 4.30 (*p* < 0.001); grip strength ↑ SMD 0.39 (*p* = 0.008) Frailty ↓ MD −2.21 (*p* < 0.001); depression ↓ MD −1.20 (*p* = 0.001) Programs >120 min/wk with aerobic + RT maximized benefit → multicomponent addresses sarco-frailty axis	AMSTAR-2: Moderate
Izquierdo et al., 2021 [45]	International Conference on Sarcopenia and Frailty Research (ICFSR) consensus of RCT evidence in older adults	Evidence-based consensus/umbrella synthesis of exercise modalities	Resistance, aerobic, balance, and multicomponent training prescriptions Muscle strength, physical function, falls Safety and dose–response for frailty/sarcopenia	Progressive RT 2–3×/wk at 70–80% 1RM → significant gains in strength, power, hypertrophy Multicomponent training (RT + aerobic + balance) superior for sarcopenia and frailty Power training at 40–60% 1RM optimal for functional mobility in oldest old	AMSTAR-2: High
Wewege & Jones, 2021 [46]	13 studies (aerobic & resistance exercise); chronic musculoskeletal pain adults	Systematic review & meta-analysis of exercise-induced hypoalgesia (EIH)	Pressure pain threshold (PPT) during/after single exercise bout Aerobic vs. resistance exercise comparison Chronic pain vs. pain-free populations	Aerobic exercise elicited large EIH: Hedges g = −0.85 at exercising site Resistance exercise produced moderate EIH: g = −0.45 EIH blunted in chronic pain populations → informs dose/modality for analgesic exercise prescription	AMSTAR-2: High
Xue et al., 2024 [47]	6 RCTs; 278 Asian older adults with sarcopenia	Systematic review & meta-analysis of resistance training	Serum IL-6, TNF-α, CRP, IL-10 Subgroup analyses: training period (<12 vs. ≥12 wks), session duration Heterogeneity and sensitivity analyses	RT ↓ IL-6: WMD −0.73 pg/mL (95% CI −1.02 to −0.44, *p* < 0.001) ≥12-wk RT: IL-6 ↓ WMD −0.91 and TNF-α ↓ WMD −1.64 (*p* < 0.01) CRP, IL-10 NS overall → RT selectively lowers IL-6 in sarcopenia, duration-dependent	AMSTAR-2: High
Rufa et al., 2019 [48]	Older adults (≥60 yrs) with chronic pain; feasibility/effectiveness of PNE	Controlled experimental trial of pain neuroscience education (PNE)	Gait speed (physical function proxy) Pain-related disability (MPI, OPQ) Fear of movement (TSK-11)	PNE significantly ↑ gait speed (*p* < 0.05) Pain disability significantly ↓ post-intervention TSK-11 fear-of-movement ↓ → PNE feasible and efficacious for older adults with chronic pain	PEDro: 7/10
Abiko et al., 2025 [49]	42 older women (65–90 yrs) with chronic low-back pain; 24 PNE + PA vs. 18 biomedical-education	Quasi-randomized controlled trial (12-wk intervention)	Physical function: 30 s Chair-Stand, daily step count (accelerometry) Pain catastrophizing (PCS) and fear-avoidance (FABQ) SPPB and TUG	Chair-Stand: d = 0.88; daily steps: d = 0.87 (large effects favoring PNE + PA) PCS ↓ d = −0.87; FABQ ↓ d = −0.65 Combined PNE with physical activity superior to education alone → integrated approach for CLBP + sarcopenia	PEDro: 8/10

RCT: randomized controlled trial; PRT: progressive resistance training; RT: resistance training; RET: resistance exercise training; PA: physical activity; PNE: pain neuroscience education; EIH: exercise-induced hypoalgesia; 1RM: one-repetition maximum; SMD: standardized mean difference; MD: mean difference; WMD: weighted mean difference; CI: confidence interval; NS: not significant; TUG: Timed Up-and-Go test; SPPB: Short Physical Performance Battery; WOMAC: Western Ontario and McMaster Universities Osteoarthritis Index; VAS: visual analog scale; NRS: numerical rating scale; PPT: pressure pain threshold; IL-6: interleukin-6; IL-10: interleukin-10; TNF-α: tumor necrosis factor-alpha; hsCRP: high-sensitivity C-reactive protein; OA: osteoarthritis; KOA: knee osteoarthritis; CLBP: chronic low-back pain; ASM: appendicular skeletal muscle; SMI: skeletal muscle index; PCS: Pain Catastrophizing Scale; FABQ: Fear-Avoidance Beliefs Questionnaire; TSK: Tampa Scale for Kinesiophobia; MPI: Multidimensional Pain Inventory; OPQ: Older-Person Pain Questionnaire; DEXA: dual-energy X-ray absorptiometry; wks: weeks; ICFSR: International Conference on Sarcopenia and Frailty Research; PEDro: Physiotherapy Evidence Database scale; AMSTAR-2: A Measurement Tool to Assess Systematic Reviews 2.

## Data Availability

No new data were created or analyzed in this study. Data sharing is not applicable to this article.

## Supplementary Materials

The following supporting information can be downloaded at: https://www.mdpi.com/article/10.3390/ijms27125204/s1. Reference [20] is cited in Supplementary Materials.

## Abbreviations

The following abbreviations are used in this manuscript:

CHARLS	China Health and Retirement Longitudinal Study
hsCRP	High-sensitivity CRP
LPS	Lipopolysaccharide
PRT	Progressive resistance training
RCTs	Randomized controlled trials
RET	Resistance exercise training
SASP	Senescence-associated secretory phenotype
TLR	Toll-like receptor
